# Novel pathogenic variant in the deficiency in *ELF4*, X-linked (DEX): case report and literature review

**DOI:** 10.1186/s13023-025-04039-x

**Published:** 2025-10-23

**Authors:** Lin Zhuo, Pengfei Ma, Miaomiao Chen, Rong Cheng, Min Li, Chuanying Li, Yun Long

**Affiliations:** Department of Gastroenterology, Anhui Provincial Children’s Hospital, Wangjiang East Road No.39, Hefei, 230051 China

**Keywords:** ELF4, DEX, Congenital immunodeficiency disease, Novel variants, Clinical symptoms, Treatment

## Abstract

**Background:**

Deficiency in *ELF4*, X-linked (DEX) is a recently recognized monogenic autoinflammatory disorder and a novel type of congenital immunodeficiency. Due to the limited number of reported cases, understanding of DEX remains incomplete. In clinical settings, children with this condition are often misdiagnosed as having diseases such as Behçet’s disease or inflammatory bowel disease, due to overlapping clinical features.

**Methods:**

Genomic DNA was isolated from peripheral blood samples for genetic analysis, and genomic DNA was extracted from the peripheral blood samples of the proband’s mother for x chromosome inactivation (XCI) analysis.

**Results:**

A retrospective analysis was conducted on the clinical and genetic characteristics of a 3-year-old male patient with DEX in China. This study offers a detailed exploration of the child’s specific clinical symptoms and treatment approach, in addition to a comprehensive review of the primary clinical manifestations and genotypic traits of individuals with DEX.

**Conclusion:**

This study aims to increase clinicians’ understanding of DEX. In cases where young male patients present with recurrent fever, oral or mucosal ulcers, or repeated infections, DEX should be strongly considered. Early implementation of gastrointestinal endoscopy and genetic testing is recommended to enable a more precise and timely diagnosis, laying a solid foundation for informed treatment decisions.

## Background

 Primary Immunodeficiency and Dysregulation Disorders (PIDD) are conditions caused by monogenic variants that result in immune system deficiencies. Clinically, these disorders are typically characterized by recurrent infections, immune dysregulation, an increased susceptibility to tumors, and multi-organ involvement [1]. Deficiency in *ELF4*, X-linked (DEX), a recently identified immune disorder, is a monogenic autoinflammatory condition [2]. Pathogenic *ELF4* variants impair the effective expression of anti-inflammatory and antiviral genes [3]. Clinically, DEX presents with fever, recurrent oral ulcers, abdominal pain, bloody stools, and other manifestations that mimic conditions such as Behçet’s disease or inflammatory bowel disease, alongside significant elevations in inflammatory markers [4]. Due to the rarity of cases and a lack of comprehensive understanding, misdiagnosis of the disorder in children has been common. This study summarizes the clinical and genetic characteristics of a child diagnosed with DEX at our hospital, offering a detailed account of the primary clinical features and genotypic characteristics, with the goal of enhancing clinicians’ understanding of DEX.

## Methods

### Genetic analysis

Genomic DNA was isolated from peripheral blood samples using the Omega DNA Mini Kit. Trio-based whole-exome sequencing (Trio-WES) was carried out by Chigene (Beijing) Translational Medical Research Center. Library preparation was conducted using the xGen Exome Research Panel v2.0 (IDT, Iowa, USA), and sequencing was performed on a DNBSEQ-T7 platform (BGI-Shenzhen). Reads were aligned to the human reference genome GRCh37 with the Burrows–Wheeler Aligner (BWA). Subsequent processing—including adapter trimming, base quality calibration, coverage analysis, and variant calling—was performed using an in-house bioinformatics pipeline. Identified variants were filtered against public databases (including the 1000 Genomes Project, gnomAD, and ExAC), retaining those with a minor allele frequency below 0.05 located in coding regions or canonical splice sites. Variants were interpreted according to ACMG guidelines. Putative variants were confirmed by Sanger sequencing using custom-designed primers

### X-chromosome inactivation (XCI) analysis

Genomic DNA was extracted from peripheral blood samples obtained from the proband’s mother. The XCI ratio was determined using a methylation-sensitive restriction enzyme-based assay followed by PCR amplification of the highly polymorphic androgen receptor (AR) gene locus. Briefly, 500 ng of genomic DNA was digested overnight with the methylation-sensitive restriction enzyme HpaII or mock-treated. The digested and undigested DNA samples were then used as templates for PCR amplification of the AR locus. The PCR products were analyzed by capillary electrophoresis to determine the allele sizes and their relative peak heights. The XCI ratio was calculated based on the relative reduction in the peak heights of the two alleles in the HpaII-digested sample compared to the undigested control.

## Results

### Case report

The patient, a 3-year-old male, was admitted to our department in May 2024 with a primary complaint of “intermittent abdominal pain for one year.” The abdominal pain, which began at the age of 2, led to two visits to the general surgery department of our hospital for suspected “appendicitis.” In the emergency room, “endoscopic retrograde appendiceal cavity irrigation” was performed. On January 6, 2024, the diagnosis of “appendix perforation” was confirmed, and the patient underwent “laparoscopic appendectomy.” Postoperatively, the child continued to experience recurrent abdominal pain, along with repeated oral ulcers. There was no history of nausea, vomiting, diarrhea, or perianal ulcers. The child has not had significant weight gain. Over the past year, the patient had six episodes of respiratory tract infections, including two common infections, two mycoplasma infections, one adenovirus infection, and one episode of tonsillitis, accompanied by elevated inflammatory markers. He is the firstborn of his mother. His parents have no siblings and no notable personal or family history.

Upon physical examination at admission, the patient was found to be 100 cm tall and weighed 15 kg, with overall normal growth and development. An ulcerative lesion was noted on the left cheek mucosa, exhibiting yellowish-white exudate. Tenderness was observed in the abdomen, particularly around the umbilicus, without rebound pain or muscle rigidity. Joint examination revealed no abnormalities.

**Fig. 1:**
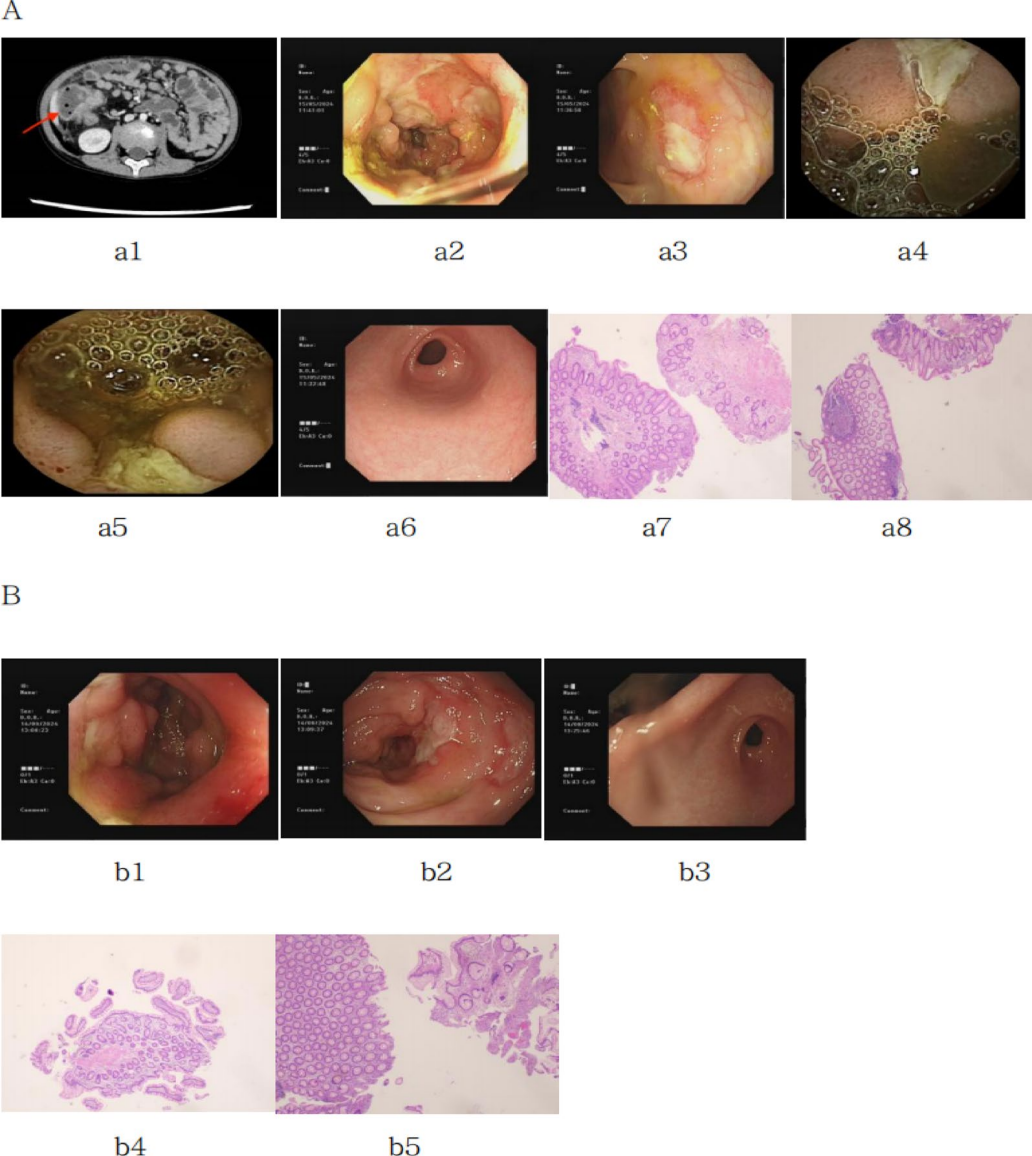
**A**: Abdominal CT, colonoscopy, gastroscopy, and pathological examination prior to treatment in the child. **a1**: Abdominal CT results show gas accumulation in the intestinal loops, with thickening and enhancement of the canal wall, indicative of inflammatory changes (indicated by arrows). **a2** and **a3**: Electron colonoscopy results reveal a “paved stone-like” appearance of the ileal mucosa, with multiple deep and large ulcers. The surrounding mucosa is swollen and fragile, prone to bleeding, and involvement of the ileocecal flap is noted. Large, deep ulcers are observed at the mouth of the ileocecal flap, with additional ulcers scattered in a diameter of approximately 0.6–1.2 cm. Multiple deep ulcers are also present in the ascending and transverse colon. **a4** and **a5**: Capsule endoscopy findings indicate multiple ulcers and stenosis in the small intestine. a6: Gastroscopy results show superficial gastritis. **a7** and **a8**: Pathological findings suggest chronic colitis, chronic ileitis, and “ileal celiac, transverse colon” large intestinal mucosa. Partial crypts are twisted and elongated, indicating potential very early-stage inflammatory bowel disease. **B**: Colonoscopy, gastroscopy, and pathological examination results after 3 months of treatment. **b1** and **b2**: Re-examination of electron colonoscopy reveals deep ulcers near the ileocecal flap at the terminal ileum, with deformation of the ileocecal flap. Deep ulcers are also observed in the ileocecal area, along with polypoid hyperplasia. Stenosis of the intestinal lumen is seen in the ascending colon, with an 11.7 mm lens body difficult to pass. Peripheral ulcers and significant mucosal swelling are present. b3: Re-examination of gastroscopy shows superficial gastritis. b4 and b5: Re-examination of pathological findings reveals eosinophil infiltration in the small intestine mucosa, with preservation of villus and crypt structures. The large intestine mucosa shows focal crypt distortion, with lesions reduced compared to previous colonoscopy biopsies

Auxiliary testing revealed an elevated white blood cell count (17.60 × 10^9^/L), neutrophil count (12.13 × 10^9^/L), C-reactive protein (CRP, 83.79 mg/L), erythrocyte sedimentation rate (33 mm/h), and fecal calprotectin (95.3 µg/g) (Table 1). Hemoglobin was low (92 g/L), and lymphocyte subset analysis showed a decreased percentage of NK cells (8.6%) and CD3 + T cells (48.5%), with a notably elevated percentage of CD3-CD19 + B cells (34.3%) (Table 2). Cytokine assays revealed elevated levels of IL-2R (1648.45 U/ml), interleukin-8 (88.61 pg/ml), and IL-6 (42.63 pg/ml) (Table 3). Immunoglobulin levels, antinuclear antibodies, anti-neutrophil cytoplasmic antibodies, cytomegalovirus、epstein-barr virus、the tests for tuberculosis infection and etiological metagenomic examination were all negative. Abdominal CT showed inflammatory changes in the right intestinal wall. Electronic colonoscopy and capsule endoscopy revealed multiple deep ulcerative strictures in the intestinal lumen. Pathology indicated a tendency toward very early-stage inflammatory bowel disease (Fig. 1).

Treatment included oral prednisone and azathioprine. As the steroids were gradually tapered, abdominal pain recurred, along with persistent elevation of inflammatory markers. After three months, the patient underwent gastroendoscopy, which revealed multiple small intestine ulcers, colon stenosis, and a risk of capsule retention, precluding further capsule endoscopy. Colonoscopy results indicated multiple intestinal ulcers with stenosis, though the pathological results suggested that the lesions had improved compared to prior examinations (Fig. 1). Given the recurrent abdominal pain, sustained elevation of inflammatory markers, and suboptimal mucosal recovery under colonoscopy, oral thalidomide was administered while tapering the steroids. Nine months after the treatment was changed, the patient is symptom-free, with normal inflammatory markers and gradual weight gain. The patient is currently undergoing close follow-up (Tables 1, 2, 3).

**Table 1:** Laboratory features of children

	First visit	One month after treatment	Three months after treatment	Six months after treatment	Nine months after treatment	Reference value
WBC(× 109/L)	17.60	6.89	15.02	7.81	5.47	4.9–12.7
N(× 109/L)	12.13	2.56	14.56	3.64	1.97	1.3–6.7
HB(g/L)	92	101	110	107	114	115–150
ESR(mm/h)	33	28	35	6	8	0–15
CRP(mg/L)	83.79	16.91	66.1	3.7	5.2	0–8
FC (ug/g)	95.3	125.9	218.5	87	43	0–50

WBC: white blood cell, N: Neutrophil number, HB: haemoglobin, ESR: erythrocyte sedimentation rate, FC: faecal calprotectin, CRP: C-reactive protein

**Table 2:** Lymphocyte subset characteristics of children

	First visit	One month after treatment	Three months after treatment	Six months after treatment	Nine months after treatment	Reference value
Percentage of CD3 + T cells	48.5	52.4	59.9	74.7	68.2	61.7–77
Percentage of CD3 + CD4 + T cells	23.6	22.5	31.8	33.4	35.2	25.8–41.6
Percentage of CD3 + CD8 + T cells	28.0	24.3	22.0	30.9	27.2	18.1–29.6
The percentage of CD3-CD16+ 56 + NK cells	8.6	9.9	3.2	7.5	6.3	10.4-19.78
Percentage of CD3-CD19 + B cells	34.3	36.4	35.3	14.9	14.7	9.02–14.1

**Table 3:** Serum cytokine characteristics of children

	First visit	One month after treatment	Three months after treatment	Six months after treatment	Nine months after treatment	Reference value
IL-1B (pg/ml)	< 5	< 5	< 5	< 5	< 5	< 5
IL-2R ( U/ml)	1648.45	942.3	1013.01	654.4	432.6	220–720
IL-8 ( pg/ml)	88.61	85.2	75.3	< 5	< 5	< 60
IL-10 ( pg/ml)	< 5	< 5	< 5	< 5	< 5	< 9
IL-6 (pg/ml)	42.63	28.6	30.2	5.52	6.10	< 7

IL: interleukin

**Fig. 2:**
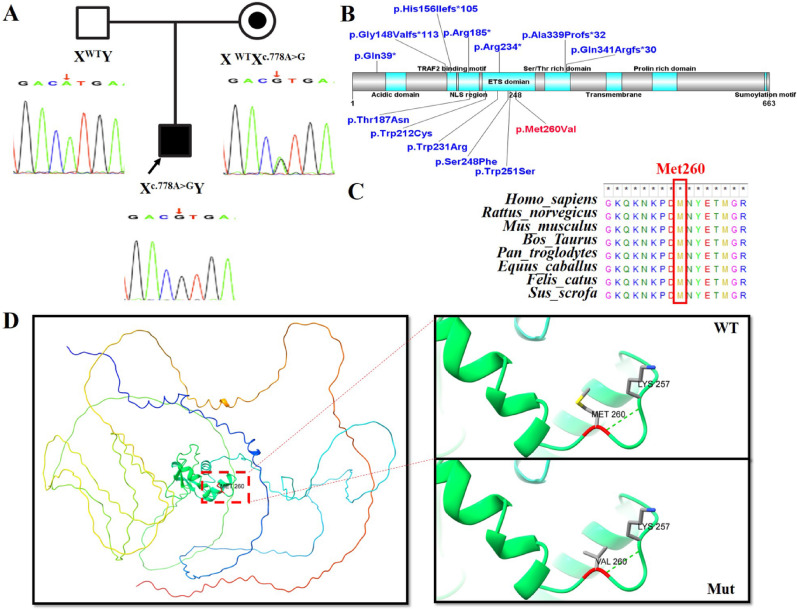
**A**: Family Pedigree (Chinese Family with X-linked *ELF4* Variant) and Sanger sequencing confirms that Proband (son) carries c.778 A > G variant in the *ELF4*, which was inherited maternally (mother is heterozygous carrier), and father has wild-type *ELF4* (no variant). Squares represent males, and circles represent females. XWT denotes the wild-type *ELF4* allele. Xc.778 A > G denotes missense variant in *ELF4*. Father (XWT Y): Unaffected wild-type; Mother (XWT Xc.778 A > G): Heterozygous carrier (asymptomatic); Son (Xc.778 A > G Y): Proband with hemizygous variant (affected). **B**: Distribution of pathogenic *ELF4* variants across functional protein domains. Illustration generated using DOG 2.0 software shows the location of reported *ELF4* variants relative to key structural domains. The novel missense variant c.778 A > G p.(Met260Val) is highlighted in red, while previously documented variants are shown in blue. **C**: Conservation analysis showing complete conservation of the identified mutated residue.D: Structural consequences of the *ELF4* p.Met260Val variant. Right: Full-length wild-type *ELF4* structure with MET260 (red) in the autoinhibitory domain. Left: Insets show detailed views of wild-type and mutant *ELF4* protein structures highlighting the c.778 A > G p.(Met260Val) substitution

Genetic exploration: A novel *ELF4* hemizygous variant, NM_001421.4: c.778 A>G p. (Met260Val), was identified. Sanger sequencing confirmed that this variant is maternally inherited (Figure 2A) (https://www.ncbi.nlm.nih.gov/gene/2000). According to the guidelines of the American Society of Medical Genetics and Genomics (ACMG) [5], the variant is classified as likely pathogenic (PM1+PM2_Supporting+PP3+PP4). The c.778 A>G p.(Met260Val) variant is located within the ETS domain, a critical functional region of the *ELF4* gene (Figure 2B). Comparative analysis of protein sequences across eight species showed high evolutionary conservation (Figure 2C). Furthermore, no benign variants have been reported in this domain among control populations. This variant is absent from population databases including dbSNP, ExAC, the 1000 Genomes Project, and gnomAD (v4.1.0). Multiple functional prediction tools indicated that the missense variant has damaging effects, as shown by Provenan (deleterious, -3.98), SIFT (damaging, 0.002), REVEL (deleterious, 0.648), Polyphen2_HDIV (possibly damaging, 0.725), Polyphen2_HVAR (possibly damaging, 0.657), M-CAP (damaging, 0.124264), MutationTaster (disease_causing, 0.99998), and CADD (Deleterious, 24.3). This variant is predicted to disrupt DNA-binding capacity, which may contribute to the disease phenotype (Figure 2D). The patient’s clinical manifestations are consistent with Deficiency in *ELF4*, X-linked (DEX).

**Fig. 3:**
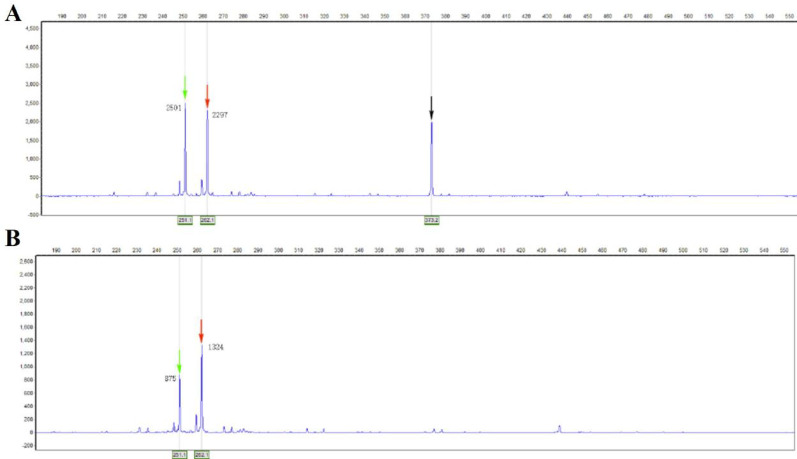
Analysis of X-chromosome inactivation (XCI) pattern in the proband’ mother using methylation-sensitive restriction enzyme digestion and fragment analysis **A**: Capillary electrophoretogram showing the amplification products of the androgen receptor (AR) gene short tandem repeat (STR) locus prior to digestion with the methylation-sensitive restriction enzyme (e.g., HpaII). The presence of two distinct peaks indicates heterozygosity at this locus, which is a prerequisite for informative XCI analysis. The X-axis represents fragment size (in base pairs) and the Y-axis indicates fluorescence intensity (in relative units). **B**: Electropherogram obtained after digestion with the methylation-sensitive enzyme. Digestion eliminates amplification from unmethylated (presumed active) X chromosomes. The ratio of the peak areas (or heights) corresponding to the two alleles was used to calculate the X-inactivation ratio. In this proband, an XCI ratio of 62% was calculated, indicating a random pattern of X-chromosome inactivation (< 70% skewed inactivation), as the derived ratio falls below the threshold commonly used to define significant skewing

X-chromosome inactivation (XCI) analysis: The analysis revealed an X-chromosome inactivation ratio of 62%, indicating a random X-inactivation pattern (Fig. 3). No significant skewing was observed based on the adopted threshold (< 70% for random inactivation). Electropherograms displayed clear amplification products before and after enzyme digestion, confirming successful experimental processing. These findings suggest that the proband does not exhibit skewed X-chromosome inactivation within the detection limits of this assay. The proband’s mother is an asymptomatic heterozygous carrier, and his father bears the wild-type allele. XCI analysis of the mother revealed a random pattern (ratio: 62%), which likely explains her lack of symptoms despite carrying the variant.

### Review of literature

**Fig. 4:**
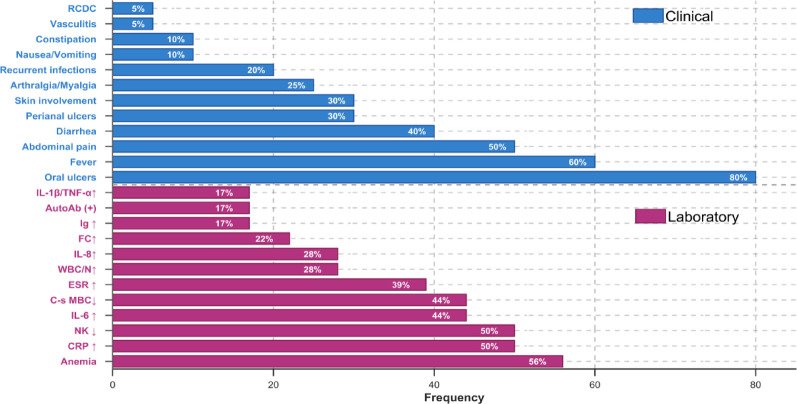
Clinical manifestations and laboratory tests of all the pediatric patients. RCDC: recurrent C. difficile colitis, IL: interleukin, TNF: tumor necrosis factor, AutoAb: autoantibody, Ig: immunoglobulins, FC: fecal calprotectin, WBC: white blood cells, N: neutrophil count, ESR: erythrocyte sedimentation rate, C-s MBC: class-switched memory B cells, CRP: C-reactive protein. Skin involvement included scattered red, tender papulonodular lesions with overlying scales, pathergy phenomenon, and skin pustules that progressed to draining ulcers

**Fig. 5:**
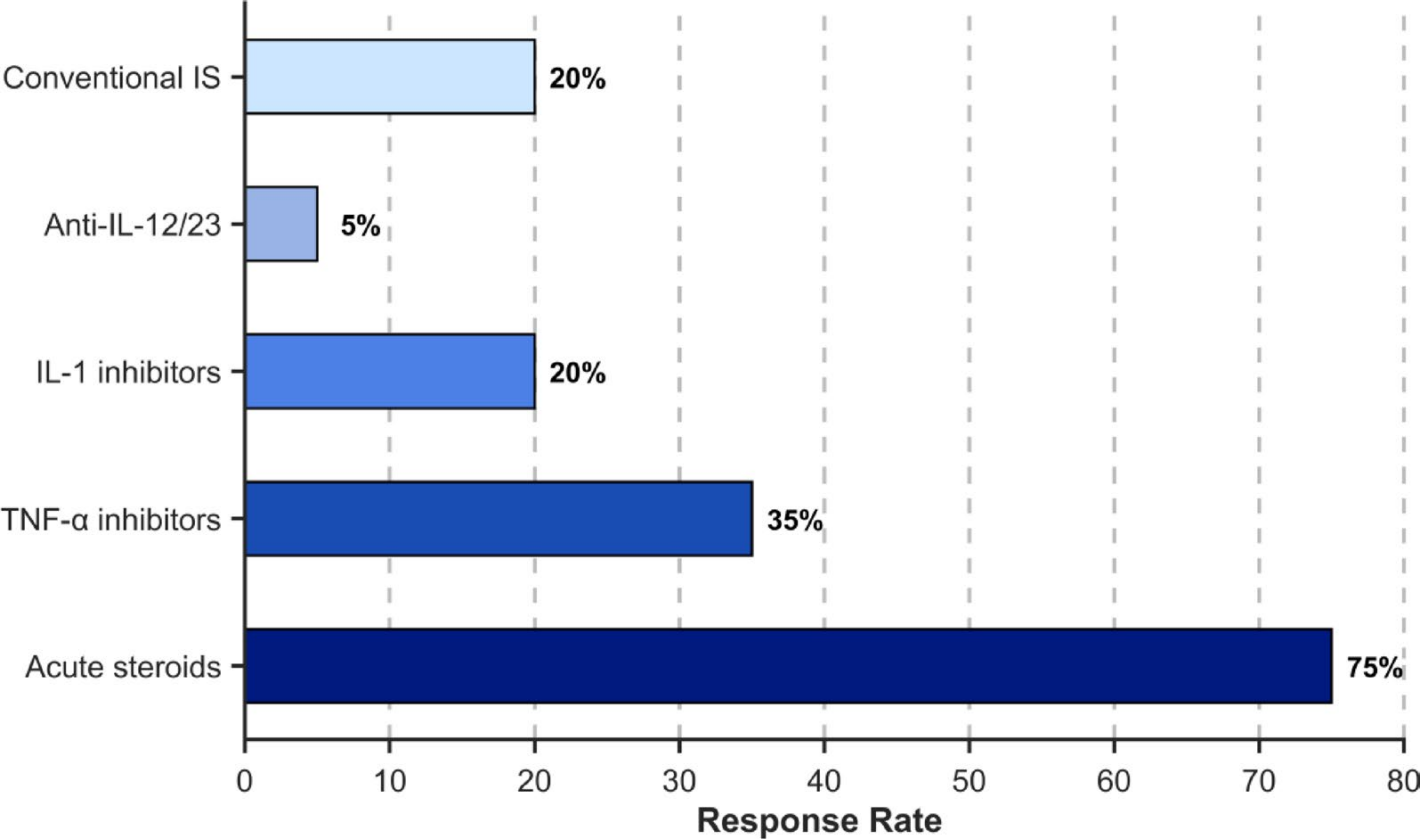
Treatment methods Conventional IS: conventional immunosuppressants (e.g., azathioprine, thalidomide, methotrexate)

A systematic review of all 20 reported male patients with confirmed DEX revealed a distinct clinical phenotype [2, 3, 6–10], with onset ages ranging from 20 days to 13 years, and 13 cases presenting before the age of 5. The most common manifestations were oral ulcers (80%, 16/20), fever (60%, 12/20), and abdominal pain (50%, 10/20), followed by diarrhea (40%, 8/20), perianal ulcers (30%, 6/20), and skin involvement (30%, 6/20). Arthralgia/myalgia (encompassing arthritis and/or myalgia) was observed in 25% (5/20), while recurrent infections (respiratory, fungal) were documented in 20% (4/20). Less frequent features included nausea/vomiting (10%, 2/20), constipation (10%, 2/20), vasculitis (5%, 1/20), and recurrent C. difficile colitis (5%, 1/20). Onset predominantly occurred before age 5 (65%, 13/20). Laboratory evaluations (*n* = 18) frequently identified anemia (56%, 10/18), elevated CRP (50%, 9/18), decreased NK cells (50%, 9/18), elevated IL-6 (44%, 8/18), decreased class-switched memory B cells (44%, 8/18), and elevated ESR (39%, 7/18). Other notable findings included elevated WBC/neutrophils (28%, 5/18 each), elevated IL-8 (28%, 5/18), elevated fecal calprotectin (22%, 4/18), elevated immunoglobulins (17%, 3/18), autoantibody positivity (17%, 3/18, including one SLE diagnosis), and elevated IL-1β/TNF-α (17%, 3/18 each). Endoscopic findings (*n* = 15) revealed intestinal ulcers in 73% (11/15) (Fig. 4). Treatment responses indicated that acute steroid therapy alleviated symptoms in 75% (15/20), while biologics (TNF-α inhibitors: 7 cases; IL-1 inhibitors: 4 cases; anti-IL-12/23: 1 case) and conventional immunosuppressants (e.g., azathioprine, thalidomide, methotrexate) were frequently effective for maintenance (Fig. 5). Following treatment, symptoms were alleviated in 11 children (55%, 11/20), 5 cases were unknown (25%, 5/20), 2 cases showed effective treatment (10%, 2/20), 1 case was effective but the medication was discontinued (5%, 1/20), and 1 case had poor treatment response (5%, 1/20). Genetically, the majority of published variants (65%, 13/20) were truncating loss-of-function (LoF) types (nonsense: 5, frameshift: 5, splice-site: 1, exon deletions: 2), while missense variants accounted for a minority (35%, 7/20). This indicates that haploinsufficiency is likely the primary disease mechanism for DEX. Notably, the recurrent R234X variant was present in 3 patients (Table 4).

**Table 4:** Characteristics of reported children with DEX

Patient	sex	Onset age	Family history	Endoscopy	Variants	Outcome
P1	Male	2y	NA	Colonoscopy: ulcers are scattered throughout the colon	c.752G > C p.(W251S)	Symptoms alleviated
P2	Male	13y	NA	NA	NM_001421.4:c.1015del p.(Ala339ProfsTer32)	effective
P3	Male	10y	NA	Colonoscopy: Isolated ulcers of distal ileum, ileocecal ileum, rectum	c.700 C > T p.(R234*)	It worked very well and was no longer used
P4	Male	5y	Mom has a history of Behçet’s disease	NA	c.115 C > T p.(Q39*)	Symptoms alleviated
P5	Male	9y	NA	Gastroscopy: chronic gastritis, esophagitis. Colonoscopy: single terminal ileum ulcer, perianal abscess, cecal lymph node hyperplasia.	c.752G > C p.(W251S)	effective
P6	Male	2 m	NA	NA	c.691T > C p.(W231R)	Symptoms alleviated
P7	Male	1y	NA	Gastroscopy: ulcers of the esophagus and antrum Colonoscopy: multiple colonic ulcers with ascending colonic stenosis, mild inflammation of colon mucosa with lymphoid hyperplasia.	NM_001421.4:c.465del p.(His156IlefsTer105)	Symptoms alleviated
P8	Male	20d	NA	Gastroscopy: gastric antrum ulcer Colonoscopy: multiple ulcers of the ileum	c.553 C > T p.(R185*)	Symptoms alleviated
P9	Male	4y	NA	NA	c.743 C > T p.(S248F)	Symptoms alleviated
P10	Male	3y	NA	Gastroscopy: gastritis and esophagitis. Colonoscopy: Persistent ulceration of terminal ileum, cecum to rectal mucosa, single epithelioid granuloma of the right colon.	c.636G > T p.(W212C)	NA
P11	Male	2y	NA	Gastroscopy: esophagitis. Colonoscopy: normal	Deletion exons 2–7 (NA)	NA
P12	Male	10y	NA	NA	Deletion exons 2–7 (NA)	NA
P13	Male	9y	Mother and grandmother had recurrent oral ulcers	Gastroscopy: gastritis. Colonoscopy: multiple ulcers in ileocecal region	NM_001421.4:c.1022del p.(Gln341ArgfsTer30)	Remission of symptoms Other clinical indicators improved
P14	Male	3.5y	NA	Gastroscopy: gastritis, esophagitis, and oral disease Colonoscopy: the terminal ileum and colonic mucosa were erythema and brittle, and aphthus-like lesions were seen.	c.700 C > T p.(R234*)	NA
P15	Male	0	NA	NA	NM_001421.4:c.443del p.(Gly148ValfsTer113)	NA
P16	Male	3 m	NA	NA	c.799 C > T, p.Arg267Trp	Symptoms are difficult to rel- ieve and rep- etitive
P17	Male	3y	NA	Colonoscopy: indicated anastomosis and anastomosis Multiple irregular, striated, deep chisel ulcers can be seen on the distal ileum Covered with white moss.	c. 248-7G > A	Symptoms alleviated
P18	Male	10y	NA	Colonoscopy: Distal ileum and ileocecal area showed ulcers	p.R234X c.(700 C > T)	Symptoms alleviated
P19	Male	3y	Mother had recurrrent oral ulcers	Gastroscopy: Esophageal ulcers, gastric congestion erosion Colonoscopy: The ascending colon is narrow and multiple ulcers can be seen in the intestine	(exon5:c.465del; XLR)	Symptoms alleviated
P20 (this report)	Male	2y	NA	Gastroscopy: Superficial gastritis Colonoscopy: There were deep ulcers in ileocecal, ascending, and transverse colon, and intestinal stenosis in ileocecal and ascending colon Capsule endoscope: Multiple ulcers of the small intestine	c.778 > G p.(Met260Val)	Symptoms alleviated

NA: not available, y: years, m: months

## Conclusion

The *ELF4* gene encodes a 663-amino acid ETS-family transcription factor essential for immune regulation [11]. Beyond its established functions in modulating CD8 + T cells [12], inhibiting Th17 cell differentiation [13], regulating NK cell function and perforin expression [14], and controlling IL-8 and MMP-9 transcription [15, 16], *ELF4* plays a critical role in innate antiviral immunity and type I interferon (IFN) production. *ELF4* directly promotes the transcription of key components in the type I IFN pathway and interferon-stimulated genes (ISGs). It also maintains immune homeostasis by promoting the expression of the anti-inflammatory IL-1 receptor antagonist (IL-1RA) and suppressing the pro-inflammatory receptor TREM1 [17, 18]. Thus, *ELF4* acts as a multifaceted negative regulator of inflammation.

Loss-of-function (LOF) variants in *ELF4* disrupt this delicate balance, leading to the autoinflammatory and immunodysregulatory phenotype seen in DEX. Defective *ELF4* function impairs type I IFN responses, compromising antiviral defense and potentially contributing to recurrent infections [17, 18]. Simultaneously, the inability to sustain IL-1RA expression and suppress TREM1 induces a heightened pro-inflammatory state [17, 18]. This dysregulation is further aggravated by impaired control over Th17 differentiation [13] and reduced ILC3 numbers and function [19], which collectively drive mucosal inflammation and neutrophil infiltration. Additionally, LOF variants cause severe defects in NK and B-cell development and function, disrupting cytotoxic responses and antibody production, thereby increasing susceptibility to recurrent infections and hematologic malignancies [14].

 Clinically, the molecular pathogenesis of DEX manifests as the hallmark features of fever, mucosal ulceration (oral, gastrointestinal, perianal), skin lesions, arthritis, vasculitis, recurrent infections, and elevated systemic inflammatory markers (Table 4). *ELF4*’s inhibition of Th17 differentiation is particularly important, as Th17 cells are key drivers of mucosal inflammation and neutrophil recruitment in both humans and mice [20]. *ELF4* also plays a pivotal role in maintaining mucosal integrity, as shown in ELF4 knockout mice [19]. Moreover, blocking TREM1 with a fusion protein in mice significantly alleviated colitis, while ELF4 knockout mice exhibited exacerbated intestinal inflammation and a marked reduction in ILC3 cell number and function [19]. In DEX, the dysfunction of immunosuppressive molecules such as IL1RN prevents the resolution of infection-induced inflammation, leading to persistent inflammation and the manifestation of chronic inflammatory symptoms like oral ulcers, intestinal ulcers, and arthritis. This ongoing inflammation intensifies fever, abdominal pain, and diarrhea. The severe developmental and functional defects in NK and B cells seen in DEX further predispose individuals to recurrent infections and increase the risk of hematologic malignancies.

The treatment of patients with DEX should be personalized according to each patient’s clinical presentation, with a focus on ongoing assessment of intestinal inflammation, oral ulcers, skin involvement, and joint pain. Current treatment strategies generally align with those used for inflammatory bowel disease and Behçet’s disease. Short-term steroid therapy can provide symptomatic relief, but biologic agents are recommended to induce disease remission and reduce reliance on steroids. For maintenance therapy, agents such as aminosalicylates, azathioprine, methotrexate, colchicine, thalidomide, and calcineurin inhibitors have shown varying degrees of success. TNF-α inhibitors (infliximab, adalimumab) are typically considered first-line biologic therapies, while ustekinumab (anti-IL-12/23) may be considered in cases where TNF-α inhibitors are ineffective [21]. Available reports suggest that TNF-α inhibitors effectively control fever and intestinal lesions in most children with DEX. Monitoring of ESR and/or CRP levels is useful for assessing disease severity, and fecal calprotectin levels can be employed to track disease activity and evaluate treatment response [22, 23]. In the current case, the patient initially received steroid therapy and azathioprine, but symptoms recurred during steroid tapering. Thalidomide was then added to the treatment regimen, helping to control inflammatory markers and alleviate intestinal symptoms. In the event of further symptom recurrence, biologic-based combination therapy will be considered. Given the complex pathogenesis of DEX, which likely involves multiple signaling pathways, alternative treatment strategies—such as bone marrow stem cell transplantation, which has induced remission in other refractory immunodeficiencies [24]—warrant exploration for children with poor responses to conventional immunosuppressants and biologics.

A confirmed case of DEX was reported in this study, with significant improvement observed following immunosuppressive treatment. Although the follow-up period remains short, continuous monitoring of the child’s progress is essential for a thorough evaluation of treatment efficacy. Ongoing data collection and analysis will be conducted throughout the follow-up process.

The p.(Met260Val) variant identified in our patient resides within the critical ETS DNA-binding domain of *ELF4*. Functional studies on other missense variants within this domain, such as p.(Trp231Arg) reported by Sun et al. (2022), have demonstrated loss-of-function effects through impaired protein stability, abolished transactivation activity, and defective binding to target gene promoters [3]. Although functional validation for the p.(Met260Val) variant was not performed in this study due to resource constraints, its location within the same functional domain, high conservation, damaging in silico predictions, and co-segregation with disease strongly support its pathogenic role through similar mechanisms.

Additionally, a systematic review and detailed analysis of the clinical characteristics of 20 patients with confirmed DEX were performed. This comprehensive examination of clinical manifestations, laboratory results, and disease progression is intended to provide healthcare professionals with valuable clinical insights. The goal is to enhance understanding of DEX, enabling more accurate diagnoses and more effective treatments in future clinical practice.

Ongoing research into the molecular mechanisms of *ELF4* variants and their role in DEX pathogenesis is gradually clarifying the origins of the clinical manifestations in patients. This critical discovery opens new avenues for DEX treatment, offering the potential to develop more targeted and effective therapeutic strategies, potentially improving patient outcomes. Additionally, while bone marrow stem cell transplantation remains a potential treatment option, its feasibility and clinical efficacy in DEX require further investigation. More basic research and clinical trials are urgently needed to assess its clinical applicability and future potential.

## Data Availability

Data will be made available upon reasonable request.

## References

[1] Fischer A. Human primary immunodefciency diseases: a perspective. Nat Immunol. 2004;5:23–30. 14699405 10.1038/ni1023

[2] Tyler PM, Bucklin ML, Zhao M, et al. Human autoinflammatory disease reveals ELF4 as a transcriptional regulator of inflammation. Nat Immunol. 2021;22(9):1118–26. 10.1038/s41590-021-00984-4. 34326534 10.1038/s41590-021-00984-4 PMC8985851

[3] Sun G, Qiu L, Yu L, et al. Loss of function mutation in ELF4 causes autoinflammatory and immunodeficiency disease in human. J Clin Immunol. 2022;42(4):798–810. 10.1007/s10875-022-01243-3. 35266071 10.1007/s10875-022-01243-3

[4] Tangye SG, Ai-herzl W, Bousfiha A, et al. Human inborn errors of immunity: 2022 update on the classification from the international union of immunological societies expert committee. J Clin Immunol. 2022;42(7):1473–507. 10.1007/s10875-022-01289-3. 35748970 10.1007/s10875-022-01289-3 PMC9244088

[5] Richards S, Aziz N, Bale S, et al. Standards and guidelines for the interpretation of sequence variants: a joint consensus recommendation of the American college of medical genetics and genomics and the association for molecular Pathology[J]. Genet Med. 2015;17(5):405–24. 10.1038/gim.2015.30. 25741868 10.1038/gim.2015.30 PMC4544753

[6] Sun G, Wu M, Lv Q, et al. A multicenter cohort study of immune dysregulation disorders caused by ELF4 variants in China[J]. J Clin Immunol. 2023;43(5):933–9. 10.1007/s10875-023-01453-3. 36823308 10.1007/s10875-023-01453-3

[7] Olyha SJ, O′Connor SK, Kribis M, et al. Deficiency in ELF4, X-linked: a Monogenic disease entity resembling behçet′s syndrome and inflammatory bowel disease[J]. J Clin Immunol. 2024;44(2):44. 10.1007/ s10875-023-01610-8. 38231408 10.1007/s10875-023-01610-8 PMC10929603

[8] Sun L, Han Y, Li B, et al. A novel frameshift variant of the ELF4 gene in a patient with autoinflammatory disease: clinical Features, transcriptomic profiling and functional Studies[J]. J Clin Immunol. 2024;44(6):127. 10.1007/s10875-024-01732-7. 38773005 10.1007/s10875-024-01732-7

[9] Zhou Yu WANG, Libo ZHANG, Chunyan. Two cases of X-linked ELF4 gene defect and literature review [J]. Chin J Pediatr. 2019;62(12):1164–8. 10.3760/cma.j.cn112140-20240425-00289 39563044

[10] Wang Nan XIE, Yongmei WANG. 2 cases of behcet’s disease like syndrome with ELF4 gene defect [J]. J Sichuan Univ (Med Edition). 2019;55(3):756–61. 10.12182/20240560606 PMC11211776 38948265

[11] SUICO MA, SHUTO T. Roles and regulations of the ETS transcription factor ELF4/MEF. J Mol Cell Biol. 2017;9(3):168–77. 10.1093/jmcb/mjw051. 27932483 10.1093/jmcb/mjw051 PMC5907832

[12] Yamada T, Park CS, Mamonkin M, Lacorazza HD. Transcription factor ELF4 controls the proliferation and homing of CD8 + T cells via the Krüppel-like factors KLF4 and KLF2. Nat Immunol. 2009;10:618–26. 19412182 10.1038/ni.1730 PMC2774797

[13] Lee PH, Puppi M, Schluns KS, et al. The transcription factor E74- like factor 4 suppresses differentiation of proliferating CD4 + T cells to the Th17 lineage. J Immunol. 2014;192(1):178–88. 24259505 10.4049/jimmunol.1301372 PMC3872250

[14] Lacorazza HD, Miyazaki Y, di Cristofano A, Deblasio A, Hedvat C, Zhang J, et al. The ETS protein MEF plays a critical role in Perforin gene expression and the development of natural killer and NK-T cells. Immunity. 2002;17:437–49. 10.1016/S1074-7613(02)00422-3. 12387738 10.1016/s1074-7613(02)00422-3

[15] Hedvat CV, Yao JJ, Sokolic RA, Nimer SD. Myeloid ELF1-like factor is a potent activator of interleukin-8 expression in hematopoietic cells. J Biol Chem. 2004;279:6395–400. 10.1074/jbc.M307524200. 14625302 10.1074/jbc.M307524200

[16] Seki Y, Suico MA, Uto A, Hisatsune A, Shuto T, Isohama Y, et al. The ETS transcription factor MEF is a candidate tumor suppressor gene on the X chromosome. Cancer Res. 2002;62(22):6579–86. 12438253

[17] You F, Wang P, Yang L, Yang G, Zhao YO, Qian F, et al. ELF4 is critical for induction of type i interferon and the host antiviral response. Nat Immunol. 2013;14:1237–46. 10.1038/ni.2756. 24185615 10.1038/ni.2756 PMC3939855

[18] Szabo A, Rajnavolgyi E. Finding a Fairy in the forest: ELF4, a novel and critical element of type I interferon responses. Cell Mol Immunol. 2014;11:218–20. 24658434 10.1038/cmi.2014.1 PMC4085491

[19] Du HQ, Zhao XD. Current Understanding of ELF4 defciency: a novel inborn error of immunity. World J Pediatr. 2024;20(5):444–50. 10.1007/s12519-024-00807-0. Epub 2024 May 11. 38733460 10.1007/s12519-024-00807-0

[20] LITTMAN D R, RUDENSKY A Y. Th17 and regulatory T cells in mediating and restraining inflammation. Cell, 2010, 140(6): 845–858. doi: 0.1016/j.cell.2010.02.021. 10.1016/j.cell.2010.02.021 20303875

[21] van Rheenen PF, Aloi M, Assa A, et al. The medical management of paediatric crohn′s disease: an ECCO-ESPGHAN guideline update[J]. J Crohns Colitis. 2020;jjaa161. 10.1093/ecco-jcc/jjaa161. 10.1093/ecco-jcc/jjaa161 33026087

[22] Sipponen T, Savilahti E, Kärkkäinen P, Kolho KL, Nuutinen H, Turunen U, et al. Fecal calprotectin, lactoferrin, and endoscopic disease activity in monitoring anti-TNF-alpha therapy for crohn’s disease. Inflamm Bowel Dis. 2008;14:1392–8. 18484671 10.1002/ibd.20490

[23] D’Haens G, Ferrante M, Vermeire S, Baert F, Noman M, Moortgat L, et al. Fecal calprotectin is a surrogate marker for endoscopic lesions in inflammatory bowel disease. Inflamm Bowel Dis. 2012;18:2218–24. 22344983 10.1002/ibd.22917

[24] Zhou Y, He L, Yu Z, et al. Reversal of SLE and hemophagocytic lymphohistiocytosis caused by lysinuric protein intolerance through allogeneic hematopoietic stem cell transplantation[J]. J Allergy Clin Immunol. 2023;151(6):1673–4. 10.1016/j.jaci.2023.03.006. 37002123 10.1016/j.jaci.2023.03.006

